# Ginsentide-like Coffeetides Isolated from Coffee Waste Are Cell-Penetrating and Metal-Binding Microproteins

**DOI:** 10.3390/molecules28186556

**Published:** 2023-09-10

**Authors:** James P. Tam, Jiayi Huang, Shining Loo, Yimeng Li, Antony Kam

**Affiliations:** 1 Synthetic Enzymes and Natural Products Center, School of Biological Sciences, Nanyang Technological University, 60 Nanyang Drive, Singapore 637551, Singapore; huan0273@e.ntu.edu.sg (J.H.); shining.loo@xjtlu.edu.cn (S.L.); leahlym@163.com (Y.L.); antony.kam@xjtlu.edu.cn (A.K.); 2 Academy of Pharmacy, Xi’an Jiaotong-Liverpool University, Suzhou 215123, China; 3 School of Food Science and Engineering, South China University of Technology, Guangzhou 510640, China; 4 Department of Biological Sciences, Xi’an Jiaotong-Liverpool University, Suzhou 215123, China

**Keywords:** cysteine-rich peptides, cell penetration, microproteins, metal-binding peptides, coffee waste product, chemical synthesis, hevein-like peptide, non-chitin binding hevein, ginsentide

## Abstract

Coffee processing generates a huge amount of waste that contains many natural products. Here, we report the discovery of a panel of novel cell-penetrating and metal ion-binding microproteins designated coffeetide cC1a–c and cL1–6 from the husk of two popular coffee plants, *Coffea canephora* and *Coffea liberica*, respectively. Combining sequence determination and a database search, we show that the prototypic coffeetide cC1a is a 37-residue, eight-cysteine microprotein with a hevein-like cysteine motif, but without a chitin-binding domain. NMR determination of cC1a reveals a compact structure that confers its resistance to heat and proteolytic degradation. Disulfide mapping together with chemical synthesis reveals that cC1a has a ginsentide-like, and not a hevein-like, disulfide connectivity. In addition, transcriptomic analysis showed that the 98-residue micrcoproten-like coffeetide precursor contains a three-domain arrangement, like ginsentide precursors. Molecular modeling, together with experimental validation, revealed a Mg^2+^ and Fe^3+^ binding pocket at the N-terminus formed by three glutamic acids. Importantly, cC1a is amphipathic with a continuous stretch of 19 apolar amino acids, which enables its cell penetration to target intracellular proteins, despite being highly negatively charged. Our findings suggest that coffee by-products could provide a source of ginsentide-like bioactive peptides that have the potential to target intracellular proteins.

## 1. Introduction

Coffee beans, frequently used to make beverages and food products, are the second-largest commodity worldwide and belong to the *Rubiaceae* family [1]. However, various parts of coffee plants have ethnomedicinal uses [2,3]. For example, seed decoction has been used to treat influenza and to increase milk production for nursing mothers, whereas leaf decoction has been used for treating anemia, edema, asthenia, and rage [4,5,6]. Coffee-fruit decoction can be used for treating hepatitis and as a stimulant for sleepiness, drunkenness, and antitussive in flu and lung ailments [4,5,6]. Coffee is a known antioxidant [7,8] and displays certain benefits for cardiovascular diseases [9,10]. Thus far, reported bioactive compounds are small-molecule metabolites such as caffeine [11,12,13], diterpene [14,15], and chlorogenic acid [16,17,18].

Among >100 species of Coffea, *C. canephora*, also known as *C. robusta*, is the second most commonly planted species because it can reach great crop yields [19]. However, coffee processing generates about 50% of waste, mainly coffee pulp and husk [19,20]. Based on the ethnomedicinal uses of coffee plants, it is likely that bioactive compounds are present in coffee waste.

Drug-like natural products in plants include both small molecules and peptides, but peptides are under-explored [21,22,23]. A likely reason for this is that they are often susceptible to degradation in the gastrointestinal tracts. Our laboratory has focused on identifying super-compact, proteolysis-resistant cysteine-rich peptides from medicinal plants. We focused on cystine-dense microproteins with molecular weights ranging from 2–5 kDa and 3–5 disulfide bonds [24,25,26,27,28,29,30].

Heveins and hevein-like peptides (Hev) are 3–5 disulfided CRPs with an evolutionary conserved CC (adjacent cysteine) motif and a chitin-binding (CB) domain [31,32,33,34,35]. The 8C-Hevs are commonly found in plants and all major cereals as well as pseudo cereals [29,36,37]. The presence of chitin-binding domains in Hevs and Hev-like peptides confer on them a specific role in host defense because of their ability to bind to chitin, which is a major constituent of fungal walls and insect exoskeletons [38].

Recently, we identified ginsentides, a family of novel non-chitin-binding (NCB) 8C-Hevs found in three commercially important ginseng species: *Panax ginseng*, *Panax quinquefolius*, and *Panax notoginseng* [24]. Ginsentides share a similar cysteine motif to 8C-Hevs, with a tandemly connecting cysteine, but they lack a chitin-binding domain and possess a different disulfide connectivity [24]. Thus far, only ginsentides, which are cell-penetrating microproteins, have been characterized. Functionally, ginsentides could be the major active compounds responsible for the “cure-all” effect of ginseng because they coordinate multiple physiological systems to relax blood vessels and relieve stress [39,40].

Herein, we report on the isolation, identification, and characterization of a panel of novel ginsentide-like peptides, designated coffeetides cC1a–c cL1a–c and cL2–6 from the pulps and husks of the coffee processing by-products from *C. canephora* and *C. liberica* of the *Rubiaceae* family. A combination of proteomic and transcriptomic methods was used to identify and validate the sequences of coffeetides with a cysteine motif similar to 8C-Hevs and ginsentide families. Its ginsentide-like disulfide connectivity and structure were confirmed by disulfide mapping, NMR analysis, and then total chemical synthesis. Furthermore, biosynthetic analysis showed that coffeetides adopt a three-domain precursor sequence with a signal peptide, pro-peptide, and a mature peptide, which is shared by the ginsentide family but it is different from chitin-binding 8C-Hev precursor arrangements. Taken together, our findings provide new insights into a new ginsentide-like microprotein family. The discovery of bioactive compounds such as coffeetides from coffee waste could help to induce incentives to reduce and recycle waste products for a sustainable environment.

## 2. Results

### 2.1. Mass-Spectrometry Screening, Isolation, and Purification of Cysteine-Rich Peptides in Aqueous Extracts of C. canephora and C. liberica

**Figure 1:**
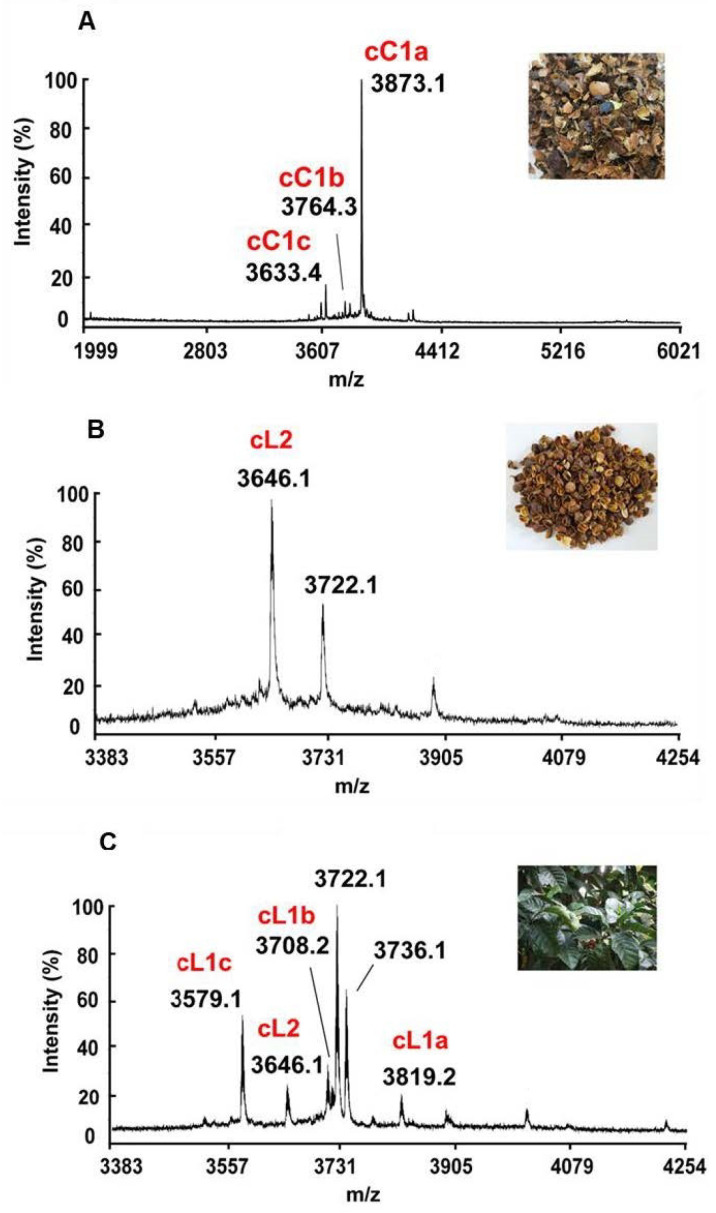
Mass spectrometry profiles of aqueous extracts of different plant parts derived from *C. canephora* and *C. liberica* using MALDI-TOF MS. (**A**) Aqueous extract of *C. canephora* husks. A cluster of peaks in the 2–6 kDa range was observed, and designated coffeetides cC1a–c; (**B**) Aqueous extract of *C. liberica* husks. A cluster of peaks in the 3.5–4.5 kDa range was observed, which we designated as coffeetide cL2; and (**C**) Aqueous extract of *C. liberica* leaves. A cluster of peaks in the 3.5–4.5 kDa range was observed, which we designated as coffeetides cL1a–c, cL2. Additional peaks at 3722.1 and 3736.1 Da are peptides the sequences of which are not found in husks and have not been characterized.

We focused on *C. canephora* and *C. liberica* because their fresh plant materials were readily available to our laboratory in Singapore. The mass spectrometry (MS) profiles of aqueous extracts of *C. canephora* and *C. liberica* revealed clusters of putative cysteine-rich peptides (CRPs) between 3500–4000 Da (Figure 1). For confirmation, we performed *S*-reduction and *S*-alkylation experiments using dithiothreitol (DTT) and iodoacetamide (IAM), respectively. The observed mass increase of 464 Da through matrix-assisted laser desorption/ionization time-of-flight mass spectrometry (MALDI-TOF MS) indicated the presence of eight cysteine residues. We named these new CRPs coffeetides (Supplementary Figures S1 and S2).

The husks of *C. canephora* and *C. liberica* were pulverized and extracted with water, filtered, and purified by C_18_ flash chromatography eluted with increasing concentrations of ethanol (20–80%). Using MALDI-TOF MS, we detected and combined the fractions containing the desired range of CRPs (2 kDa to 6 kDa) (Figure 1). These fractions were then purified by anion-exchange chromatography. Eluents containing coffeetides were pooled and purified by multiple rounds of preparative reversed phase-high performance liquid chromatography (RP-HPLC) to homogeneity and their identities were confirmed by MALDI-TOF MS (Supplementary Figures S3 and S4). Under our extraction conditions, coffeetides cC1a and cL1a were the most abundant in the husks of *C. canephora* and *C. liberica.*

### 2.2. Sequencing, Database Search, and Transcriptomic Analysis of Coffeetides

**Figure 2:**
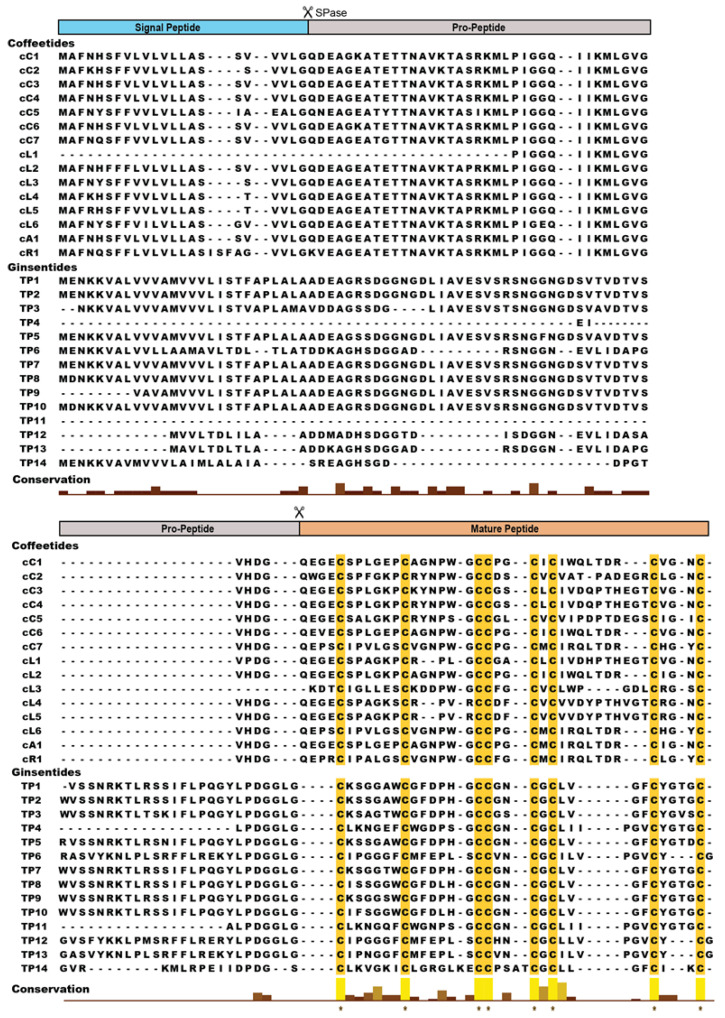
Precursor sequence alignment of coffeetides and ginsentides. Coffeetides and ginsentides have three domain precursors of a signal peptide domain, pro-peptide domain, and a mature peptide domain. The histogram depicts the conservation of amino acids in the sequences of coffeetides from *C. canephora* (cC1–cC7), *C. liberica* (cL1–cL6), *C. arabica* (cA1), and *C. racemosa* (cR1), and ginsentides from ginseng plants *Panax ginseng*, *Panax quinquefolius*, and *Panax notoginseng* (TP1–TP14). SPase: signal peptidase. The highly conserved eight cysteine residues are highlighted in yellow. * Highly conserved residues.

To determine the amino acid sequences of coffeetides and their N-terminal cleavage site of the mature peptides from their biosynthetic precursors, library-assisted LC-MS/MS sequencing was performed to identify seven coffeetide sequences. They included cC1a, cC1b, cC1c, cL1a, cL1b, cL1c, and cL2, from *C. canephora* (cC1a–c) and *C. liberica* (cL1–2) species (Figure 2).

The transcriptomic and proteomic analyses (Figure 2 and Table 1) show that the N-terminal cleavage site of most coffetides, including cC and cL, is between Gly and Gln. N-terminal Gln spontaneously cyclizes to pyroglutamine, which explains the occurrence of pyroGlu as the N-terminus of cC1a. The truncated N-terminal analogs of cC1 give rise to cC1b and cC1c.

8C-Hevs can be characterized by an evolutionarily conserved cysteine spacing pattern of CXnCXnCCXnCXnCXnCXnC with an adjoining CC at positions 3 and 4. A typical chitin-binding domain has a conserved motif of SXΦXΦ (Φ, aromatic residues; X, any amino acid) in two intercysteine loops: between CysIV and CysV and a conserved aromatic residue between the CysV and CysVI [36]. This binding site is stabilized by multiple disulfide bonds. In contrast, NCB 8C-Hevs, such as ginsentides, lack the chitin-binding domain and have a much-shortened amino sequence between CysIV and CysVI [24]. To discover additional coffeetides, we performed a database search for coffeetide sequences using ginsentide sequences. Table 1 shows the results of the database search to identify coffeetides in four coffee plants, which included *C. canephora*, *C. liberica*, *C. Arabica,* and *C. racemosa*, with molecular weights ranging from 3579 to 4234 Da. All coffeetides, like ginsentides, lack a chitin-binding domain.

### 2.3. Disulfide Mapping of Coffeetide cC1a

**Figure 3:**
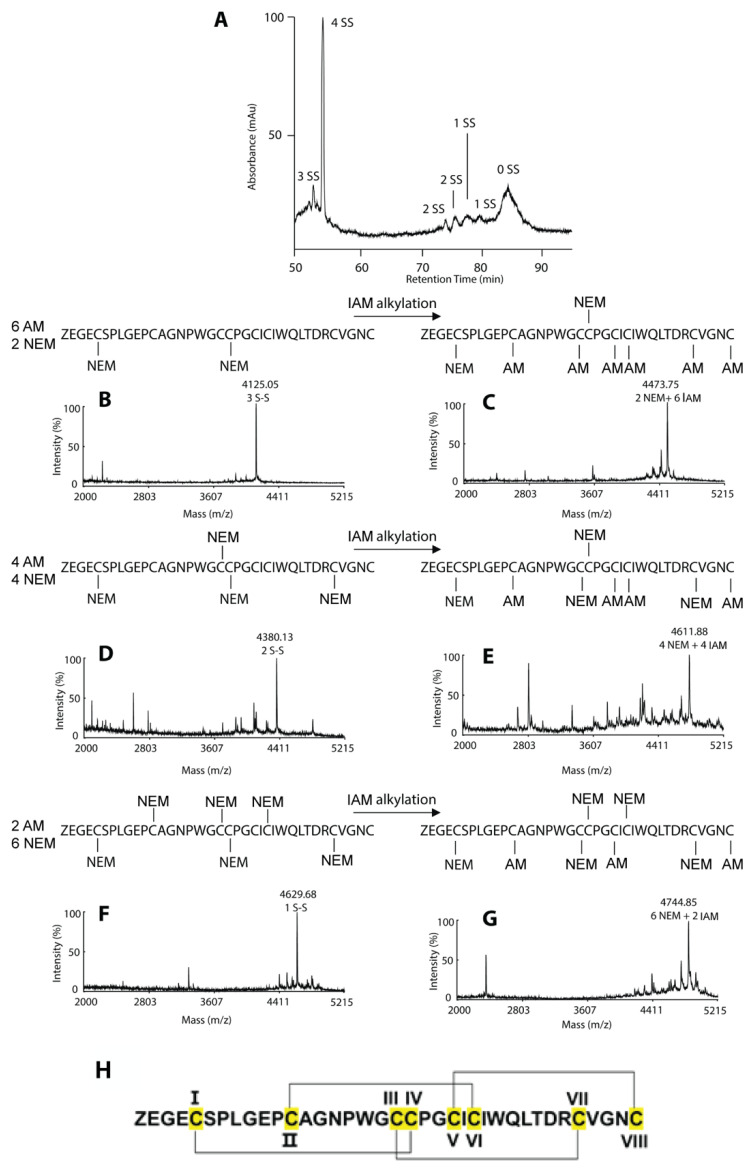
Disulfide-mapping of coffeetide cC1a. A stepwise partial reduction alkylation using Tris (2-carboxyethyl) phosphine (TCEP) and N-ethylmaleimide (NEM), and full reduction and alkylation with dithiothreitol (DTT) and iodoacetamide (IAM) were performed. The 1-SS, 2-SS-, and 3-SS-intermediates were purified using RP-HPLC and analyzed using MALDI-TOF/TOF MS/MS. (**A**) The RP-HPLC profile of the different SS-intermediates of cC1a. (**B**) MS profile of the partially reduced and alkylated 3-SS intermediate. (**C**) MS profile of the fully reduced and alkylated 3-SS intermediate. (**D**) MS profile of the partially reduced and alkylated 2-SS intermediate. (**E**) MS profile of the fully reduced and alkylated 2-SS intermediate. (**F**) MS profile of the partially reduced and alkylated 1-SS intermediate. (**G**) MS profile of the fully reduced and alkylated 1-SS intermediate. (**H**) The disulfide connectivity of coffeetide cC1a is CysI–IV, CysII–VI, CysIII–VII, and CysV–VIII.

To confirm that coffeetides indeed belong to the ginsentide family, we performed the disulfide mapping of coffeetide cC1a. Coffeetide cC1a was first partially *S*-reduced with Tris (2-carboxyethyl) phosphine (TCEP) and selectively *S*-alkylated with N-ethylmaleimide (NEM) under acidic conditions to obtain NEM-alkylated cC1a as one- (1-SS), two- (2-SS), and three- (3-SS) disulfide species. They were purified by reversed-phase HPLC (Figure 3). The purified intermediates were then subjected to another round of reduction by DTT and *S*-alkylation by a second *S*-alkylating reagent, iodoactamide (IAM), under basic conditions. Each intermediate was subjected to sequencing by MALDI-TOF/TOF MS/MS. The 3-SS intermediate revealed the CysI–IV disulfide linkage, the 2-SS the CysIII–VII, and the 1-SS CysII–VI. The fourth disulfide bond, CysV–VIII, was obtained by deduction. Put together, coffeetide cC1a shares the same disulfide connectivity as ginsentides (Figure 3). Furthermore, we confirmed the disulfide connectivity of coffeetide cC1a by 2D-Nuclear Magnetic Resonance (NMR) (Supplementary Figure S5). Thus far, this pattern of disulfide connectivity is only unique to ginsentides [24].

### 2.4. Chemical Synthesis and Oxidative Folding of Coffeetide cC1a

To prepare a sufficient quantity of coffeetide cC1a for characterization, we performed its synthesis using solid phase synthesis. Synthetic coffeetide cC1a was used for the remaining studies unless stated otherwise. We used a stepwise, Fmoc(fluorenylmethyloxycarbonyl)-tert-butyl protecting group strategy to assemble the cC1a sequence on resin supports and an acidic cleavage to release the linear synthetic coffeetide cC1a precursor and their protecting groups (Supplementary Figure S6). RP-HPLC was performed to purify the synthesized peptide precursor and MALDI-TOF MS to confirm its identity.

**Figure 4:**
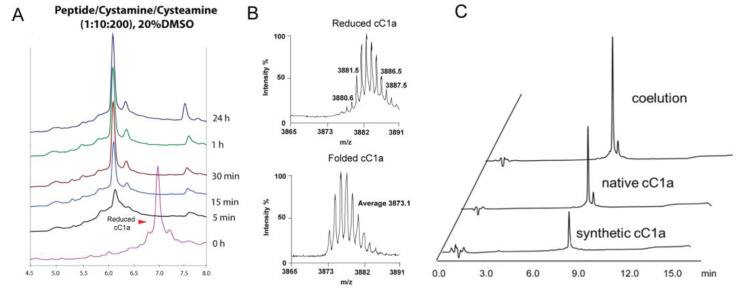
Optimized folding condition of synthetic cC1a. (**A**) Optimized folding condition for coffeetide cC1a: 0.1 M of ammonium bicarbonate buffer (pH 8.0) containing 10 mM cystamine, 200 mM cysteamine, and 20% (*v*/*v*) DMSO, incubated for 3 h at room temperature. Under the optimized folding condition, a folding yield of 82% was obtained. (**B**) MS profile of reduced and folded synthetic cC1a. (**C**) RP-UHPLC profile of the co-elution of native and synthetic cC1a.

To form all three disulfides of synthetic cC1a, we used a global oxidative approach with the assistance of dimethyl sulfoxide (DMSO) to minimize aggregation during the oxidation process. To optimize oxidative folding, we designed 18 conditions by varying concentrations of co-solvents, redox reagents, and incubation times (Run 1–18, Supplementary Table S1). First, we compared the use of redox pair cysteamine/cystamine vs. reduced and oxidized glutathione (GSH:GSSG). We observed that the redox pair cysteamine:cystamine (100:10 mM) produced a higher yield (18%) than GSH:GSSG, which gave a 9% yield. Under these two conditions, precipitations were observed during the folding process, resulting in a low yield. Thus, we introduced DMSO in the folding solution. We observed that adding 10% and 20% DMSO to the folding reaction improved the folding yield to 59% (Run 3) and 85% (Run 4), respectively. However, no significant changes were observed when the concentration of DMSO was raised to 30% (Run 5). Previous studies have shown that isopropanol, as a co-solvent, can help in the solubility of hydrophobic CRPs [30]. In our studies, however, including 20% and 30% of isopropanol in the reaction mixture resulted in a decreased folding yield of 29% (Run 6) and 11% (Run 7), respectively, suggesting that iPrOH might hinder the oxidative folding process [30]. In Run 8–18 (Supplementary Table S1), the effects of different ratios of redox reagents and folding times were evaluated. Increasing the concentration of cystamine from 10 mM to 20 mM for a more oxidizing condition decreased the folding yield to 77% (Run 8–9). On the contrary, increasing the concentration of cysteamine from 100 mM to 200 mM to give a highly reducing condition led to a higher folding yield of 82% (Run 10–12). When cysteamine concentrations were further increased to 300 mM and 400 mM, or the reaction time was prolonged to >3 h, no significant increase in the folding yield (Run 13–18) was observed. The folding process of selected folding conditions is shown in Supplementary Figure S7, which shows the folding process of selected folding conditions. Together, our results showed that the optimized folding conditions for coffeetide cC1a require a high-reducing environment and DMSO as a co-solvent to minimize the aggregation of misfolded products (Figure 4). The optimized folding conditions were as follows: 0.1 M of ammonium bicarbonate buffer (pH 8.0) containing 10 mM cystamine, 200 mM cysteamine, and 20% (*v*/*v*) DMSO, incubated for 3 h at room temperature (Figure 4A). Under the optimized folding conditions, we obtained a folding yield of 82%. Using Reversed Phase-Ultra High-Performance Liquid Chromatography (RP-UHPLC) and NMR spectroscopy, we confirmed that the synthetic and native coffeetide cC1a are indistinguishable (Figure 4C and Supplementary Figure S5).

### 2.5. Solution NMR Structure of cC1a

**Figure 5:**
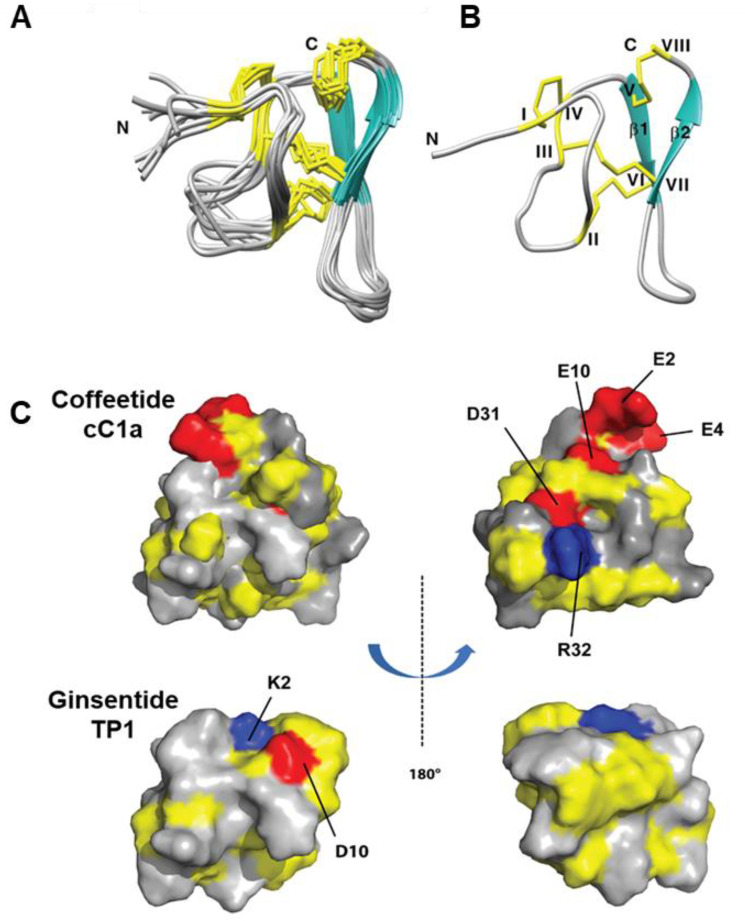
NMR structure of coffeetides cC1a and ginsentides TP1. (**A**) Superposition of the cC1a backbone traces from the final 10 ensemble solution structures and restrained energy minimization. (**B**) Ribbon representation of cC1a structure with disulfide bonds formed between CysI–CysIV, CysII–CysVI, CysIII–CysVII, and CysV–CysVIII. (**C**) Electrostatic surface comparison of cC1a (PDB: 6JI7) and ginsentide TP1 (PDB: 2ml7) in two views. The negatively charged residues (D, E), positively charged residues (K, R), and hydrophobic residues are highlighted in red, blue, and yellow, respectively.

The NMR solution structure of cC1 (Figure 5) was determined using a combination of distance restraints obtained from 2D 1H-1H-TOCSY and NOESY experiments, as well as hydrogen bond restraints derived from H/D exchange NMR experiments (Supplementary Table S2). Nearly all spin-spin systems in cC1 were identified, with the exception of the initial residue, pyroglutamine. Approximately 98% of proton resonances were unambiguously assigned. The solution structure of cC1 was established based on 216 NMR-derived distance restraints and four hydrogen bonds.

Figure 5A displays the NMR ensemble consisting of the 10 lowest-energy cC1 structures. The root-mean-square deviation (RMSD) value for the 10 most favorable structures, considering residues Gly3-Glu10 and Gly18-Cys37, was 1.10 ± 0.21 Å, while for all heavy atoms, it was 1.60 ± 0.25 Å (Supplementary Table S3). The cC1 structure is characterized by the presence of two anti-parallel small β-strands (β1: Cys25-Trp27 and β2: Cys33-Gly35), as well as several compact turns and loops.

Remarkably, the NMR-derived structure of coffeetide cC1 shares a structural fold and disulfide connectivity pattern similar to that of ginsentides [24]. Specifically, cC1 exhibits three disulfide bonds (Cys I-IV, II-VI, and III-VII) that form a characteristic cystine-knot fold. Additionally, an extra penetrating disulfide bond, Cys V-Cys VIII, connects the C-terminus to the β1 sheet. The structural conformation of cC1 is well-defined through numerous medium- and long-range NOEs (Supplementary Table S2).

The 3D structure of coffeetide cC1 has been deposited in the Protein Data Bank under the accession number 6JI7. Figure 5C provides a comparative representation of the electrostatic surface topology between cC1 and ginsentide TP1 (PDB: 2ml7) from two perspectives. Notably, when compared to TP1, coffeetide cC1 exhibits a high charge density, featuring four acidic residues (Glu2, Glu4, Glu10, and Asp31) and one basic residue (Arg32).

### 2.6. Coffeetide cC1a Is Resistant to Heat, Acid, Proteolytic, and Human Serum-Mediated Degradation

**Figure 6:**
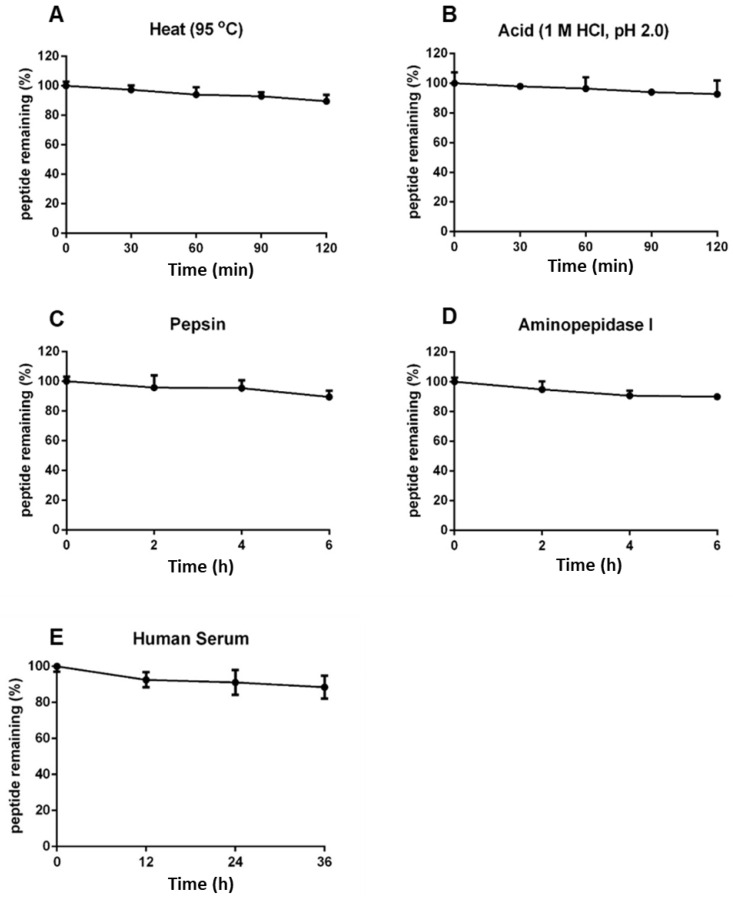
Stability assays of coffeetide cC1a. An amount of 200 µM coffeetide cC1a was subjected to (**A**) heat at 100 °C for 2 h, (**B**) acidic conditions in 1 M HCl (pH 2.0) for 2 h, (**C**) pepsin in 100 mM sodium citrate buffer (pH 2.5) for 6 h, (**D**) aminopeptidase I in 20 mM tricine for 6 h, and (**E**) human serum for 36 h. MALDI-TOF MS was used to characterize coffeetide cC1a and reversed-phase ultra-high pressure liquid chromatography was used to quantify cC1a in the treated samples to calculate the percentage of cC1a remaining intact.

Coffeetides have a compact cystine-braced structure like ginsentides, which has been previously shown to display high heat and proteolytic stability [24]. To determine the stability of coffeetides, we performed heat, proteolytic, acid, and enzymatic stability assays using native coffeetide cC1a. We determined that >88% of coffeetide cC1a remained intact, as determined by RP-UHPLC, under high heat (95 °C) or acidic conditions (pH 2) for 2 h (Figure 6A,B). Under proteolytic conditions using pepsin and aminopeptidase for 6 h, >90% coffeetide cC1a remained intact (Figure 6C,D). Similarly, >85% of coffeetide cC1a remained after being treated with human serum for 36 h (Figure 6E). Collectively, coffeetide cC1a was highly stable against heat, acid, proteolytic, and human serum-mediated degradation.

### 2.7. Coffeetide cC1a Is Non-Cytotoxic

**Figure 7:**
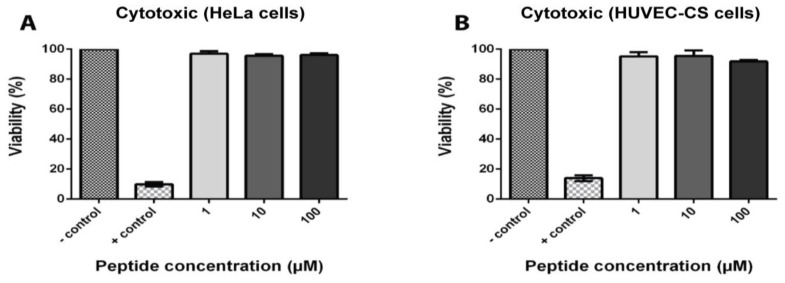
Cytotoxic activity of coffeetide cC1a on (**A**) HeLa cells and (**B**) HUVEC-CS cells. Triton X-100 was used as a positive control. All results are expressed as mean ± S.E.M. (n = 3).

To determine the toxicity of coffeetide cC1a, HeLa and HUVEC-CS cell lines were treated with different concentrations of coffeetide cC1a. Triton X-100 was used as the positive control. Figure 7 shows that coffeetide cC1a is not cytotoxic to either cell lines at concentrations up to 100 µM.

### 2.8. Coffeetide cC1a Is Cell-Penetrating

**Figure 8:**
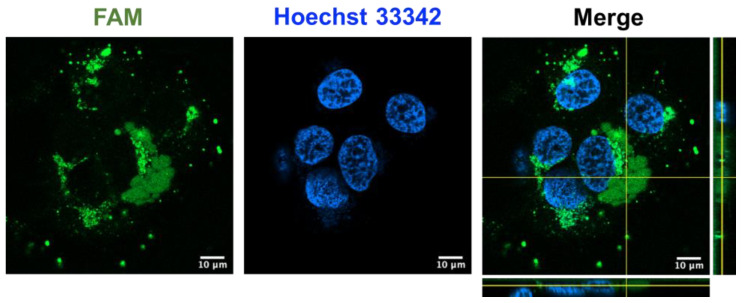
Confocal Z-stack images of fluorescent labeled FAM-cC1a in HUVEC-CS cells. 1 µM fluorescent labeled coffeetide cC1a (FAM-cC1a) (green) was incubated with HUVEC-CS cells nuclear-stained with Hoechst 33342 (blue) at 37 °C for 4 h.

Recently, our laboratory reported certain cystine-dense microproteins, including ginsentides from ginseng plants and roseltides from *Hibiscus sabdariffa* [25,40,41]. To investigate if coffeetide cC1a could penetrate cells, we prepared fluorescent labeled coffeetide cC1a (FAM-cC1a) to study its effect on HUVEC-CS cells using live-cell confocal microscopy at 37 °C for 4 h. The confocal Z-stack images revealed that FAM-cC1a could penetrate cells, and enter the cytoplasm and the nucleus (Figure 8). This is interesting as cC1a is highly negatively charged and was not expected to be cell-penetrating. The presence of a continuous stretch of 19 apolar amino acids in cC1a, however, could contribute to its amphipathicity, allowing it to penetrate cells.

### 2.9. Coffeetide cC1a Is Metal Binding

**Figure 9:**
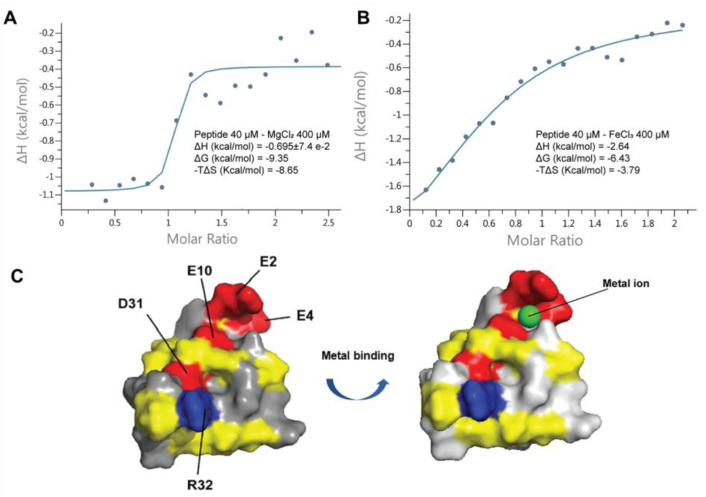
Isothermal titration calorimetry assay and proposed metal-binding pocket of cC1a. (**A**) ITC spectrum of 40 μM cC1a with 400 μM MgCl_2_, (**B**) ITC spectrum of 40 μM cC1a with 400 μM FeCl_3_. (**C**) Electrostatic surface and metal binding domain of cC1a (PDB: 6JI7). The negatively charged residues (D10, D31, E2, E4, and E10), positively charged residues (R32), and hydrophobic residues are highlighted in red, blue, and yellow, respectively.

Coffeetide cC1a is acidic, especially at its N-terminus. The solution structure of cC1a showed a three-glutamic acid cluster consisting of Glu2, Glu4, and Glu10 to form a putative metal-ion binding pocket (Figure 9). To study its metal-ion binding ability, we used an isothermal titration calorimetry (ITC) assay to determine the binding activity of cC1a with four metal ions, K^+^, Mg^2+^, Ca^2+^, and Fe^3+^. The titration results of 200 µM metal ions with cC1a at 20 °C showed that cC1a does not display reproducible binding affinity to Ca^2+^ and K^+^, but exhibits an appreciable binding affinity (K_D_) of 4.19 ± 4.79 and 1.67 ± 1.80 µM to Mg^2+^ and Fe^3+^, respectively (Figure 9). The ITC data of Mg^2+^ and Fe^3+^ fit well to a model of approximately one binding site per monomer. The ΔH and ΔG of Mg^2+^ are −0.695 ± 7.4 × 10^−2^ and −9.35 kcal/mol, respectively. The ΔH and ΔG of Fe^3+^ are −2.64 and −6.43 kcal/mol, respectively.

## 3. Discussion

The conversion of coffee waste into bioactive compounds has gained increasing attention in recent years because of its potential benefits for environmental sustainability and human health through drug discovery. Coffee waste, representing a major byproduct of the coffee industry, has routinely been disposed of through landfilling, incineration, or composting [42]. This report identifies, in coffee waste, a panel of novel coffeetides which belong to the ginsentide family. Ginsentides show high potential to treat chronic metabolic and cardiovascular diseases [39]. Our study showed that coffeetide cC1a, at its N-terminus, contains a metal-binding domain where cC1a binds to the Fe^3+^ ion. Such metal chelators are known to reduce oxidative stress by inhibiting the formation of ROS [43,44,45,46]. Furthermore, coffeetide cC1a is cell-penetrating and could potentially reduce intracellular ROS damage to maintain homeostasis. Other studies have also identified, from coffee waste, bioactive compounds with potent inhibitory effects against enzymes to improve blood glucose metabolism [47]. The discovery of these bioactive compounds provides motivation to reduce coffee waste and mitigate environmental damage.

Until now, ginsentides have represented the only family of non-chitin-binding 8C-Hevs identified from plants [24]. Coffeetides are the second to join this family of non-chitin-binding 8C-Hevs. Based on the similarity of their cysteine motif, it is difficult to distinguish ginsentides and coffeetides from 8C-Hevs. However, they can be distinguished by three other criteria, including their size, absence of the chitin-binding domain, and distinct precursor architecture.

**Figure 10:**
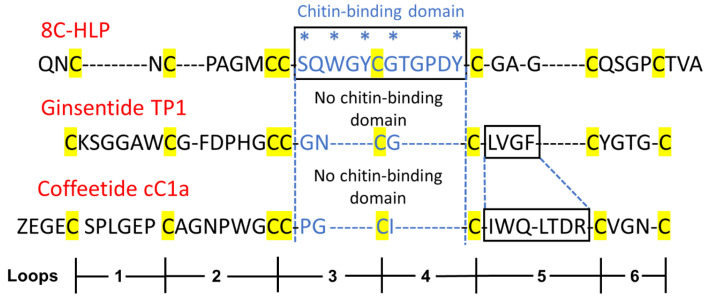
Sequence comparison of a typical 8C-Hevein-like peptide (HLP), ginsentide TP1, and coffeetide cC1a. 8C-HLP, ginsentide TP1, and coffeetide cC1a have the same conserved cysteine spacing pattern and motif (highlighted in yellow). Typical 8C-HLPs have a chitin-binding domain (* marks the highly conserved residues of the chitin binding domain of heveins and HLPs) between loops 3 and 4. Ginsentide TP1 and coffeetide cC1a lack a chitin-binding domain in loops 3 and 4.

8C-Hevs (MW around 4.3 kDa) are longer in length than ginsentides (MW around 3.1 kDa) or coffeetides (MW around 3.8 kDa). This is partly due to the lack of a chitin-binding domain in the latter two. The chitin-binding domain of Hevs and HLPs often contain >11 amino acids, whereas the number of amino acids is reduced to three in ginsentides and coffeetides, forming a new contracted motif as CCxxCxC in loops 3 and 4 (Figure 10). Coffeetides also differ from ginsentides, particularly in loop 5. Loop 5 of coffeetides is hypervariable (varying from six to eleven residues) and is longer than ginsentides, suggesting the functional plasticity of coffeetides for plant defense and adaptation.

**Figure 11:**
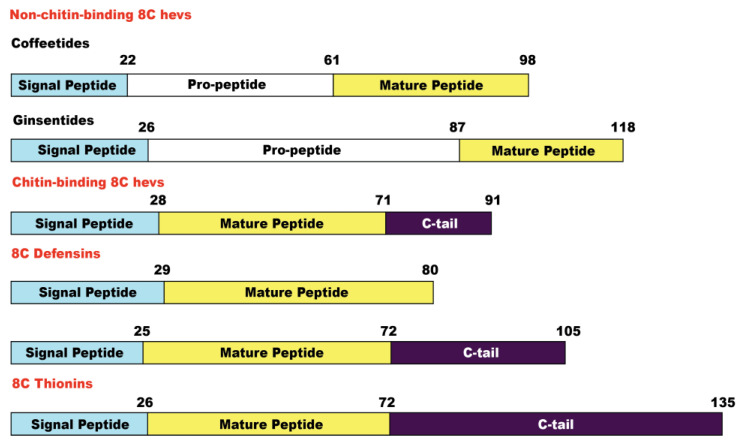
Precursor architectures of non-chitin-binding 8C Hevs (coffeetides and ginsentides), chitin-binding 8C Hevs, 8C defensins, and 8C thionins.

We used the term microproteins to name our disulfide-dense CRPs in part because their biosynthetic precursors are often <100 amino acids. Coffeetide precursors, like ginsentides, have a three-domain architecture: a signal peptide, a pro-peptide, and a mature peptide, suggesting that coffeetides undergo a secretary pathway like most plant Hevs [48]. The precursors are secreted and translocated from the cytoplasm to the endoplasmic reticulum where the signal peptide is cleaved by signal peptidase (SPase). After the cleavage and release of the pro-peptide by endopeptidase, the mature peptide is released for further post-translational modification. Figure 11 summarizes the precursor architectures of coffeetides, ginsentides, and other 8C-CRPs. They include chitin-binding 8C-Hevs, 8C-defensins, and 8C-thionins, which all have different two- or three-domain architectures of a signal peptide followed by a mature peptide, and an additional C-terminal tail. Only the non-chitin binding 8C-Hevs, coffeetides, and ginsentides, have the presence of a pro-peptide domain. The pro-peptide domain for coffeetides, however, is shorter (about 38.7 aa) compared to that of ginsentides (about 61 aa). Sequence comparison revealed that the cleavage sites between the signal peptide and pro-peptide in coffeetides are highly conserved, which are between Gly and Gln, or Lys, whereas ginsentides are between Gly and Cys (Figure 2). This feature provides insight into coffeetide biosynthesis, which is beneficial for developing a recombinant protein expression system using different organisms.

Here, we showed that coffeetides are cell-penetrating and possess high stability. These two interesting features were consistently observed, as documented in our previous reports on novel cystine-dense microproteins. Cystine-dense microproteins have a compact structure and a cystine core, which confers their high stability against proteolytic degradation, especially towards proteases commonly found in the gastrointestinal tract. Some examples include cyclotides [49,50,51,52], bradykinin-grafted in cyclotides [53], roseltide rT1 and rT7 [25,26], bleogen [28], and ginsentide [24]. Apart from their high stability, the cystine core also displaces the sidechains of hydrophobic amino acids to face outwards, creating hydrophobic surface patches and favoring cell penetration. This has been shown in our previous studies on the positively charged roseltide rT1 and the negatively charged roseltide rT7 [25,26]. Targeting intracellular proteins has drawn strong interest in recent years because the small footprints of most drugs cannot inhibit intracellular protein-protein interactions. As such, Greg Verdine has coined the phrase “drugging the undruggable” using peptides and microproteins [54]. It is worthwhile to note that our data are preliminary and warrant further studies.

In our study, we also demonstrated that coffeetide cC1a possesses metal-binding properties, particularly a strong affinity for iron ions. The cluster of N-terminal glutamic acid residues (Glu2, Glu4, and Glu10) in coffeetide cC1a is expected to create negatively charged surfaces, facilitating the formation of stable iron complexes. These complexes potentially have the ability to reduce the presence of free Fe3+ ions, thus restricting their participation in Fenton reactions that generate harmful reactive oxygen species (ROS) [55,56,57,58,59]. Iron-binding peptides, also known as iron-chelating peptides, represent a promising therapeutic strategy for managing disorders characterized by iron overload or dysregulation [60,61,62,63,64,65]. These peptides function by binding to and removing excess iron ions, effectively regulating iron homeostasis. Thus, coffeetides exhibit the potential to modulate oxidative stress by controlling iron levels and curtailing the production of reactive oxygen species (ROS), which are implicated in cardiovascular and neurodegenerative diseases [66,67,68,69]. Further studies are warranted to explore the therapeutic efficacy and safety of coffeetides in relation to oxidative stress.

In conclusion, our results show that coffeetides from coffee husks are metal-binding and cell-penetrating microproteins. They could expand leads for the design and development of metabolically stable, orally bioavailable, and cell-penetrating microproteins for biologic drugs.

## 4. Materials and Methods

### 4.1. Materials

All chemicals and solvents, unless otherwise stated, were purchased from Sigma Aldrich, St. Louis, MO, USA, and Fisher Scientific, Cleveland, OH, USA.

### 4.2. Plant Materials

*C. canephora* and *C. liberica* were collected from a local market and Nanyang Herbs Garden, Nanyang Technological University (NTU), Singapore, and authenticated by an experienced herbalist, Mr. Ng Kim Chuan, from Nanyang Herbs Garden, NTU, Singapore. A voucher specimen was deposited in the Nanyang Herbarium, School of Biological Sciences, NTU, Singapore, with the accession number CCH-20160523.

### 4.3. Extraction and Screening of Coffea canephora and Coffea liberica

Samples from *C. canephora* and *C. liberica* were initially extracted with 1 mL of water and subsequently centrifuged at 10,000× *g* for 10 min to eliminate plant debris. Then, 400 mg of ammonium sulfate was introduced into the resulting supernatant and shaken for 1 h. Following another round of centrifugation, the resultant pellet was dissolved in a solution of 10% acetonitrile (ACN) and subsequently subjected to purification using Zip-tip C18 columns (Millipore, MA, USA) prior to mass spectrometry analysis. The mass spectra of the eluted fractions were acquired using an ABI 5800 matrix-assisted laser desorption/ionization time-of-flight mass spectrometry (MALDI-TOF MS) analyzer (Applied Biosystem, Massachusetts, MA, USA).

### 4.4. Isolation and Purification of Coffeetides

*C. canephora* and *C. liberica* plant material was pulverized and subjected to water extraction at a ratio of 1:10 (*w*/*v*) at room temperature for 2 h. Following extraction, the mixture underwent centrifugation at 10,000× *g* for 20 min to eliminate plant particulates. The resulting supernatant was subsequently filtered through 1 μm and 0.45 μm pore-size filter papers and loaded onto a C18 flash column (Grace Davison, Columbia, MD, USA). Elution was performed using increasing concentrations of ethanol (20–80%). Fractions displaying a positive signal within the desired molecular weight range of 2 kDa to 6 kDa were combined for further purification. These eluents were then introduced into a flash column containing a 100 mL slurry of Q-Sepharose Fast Flow anion-exchange resin (GE Healthcare, CA, USA). The ion exchange flash column was equilibrated with a solution consisting of 5% ACN in 20 mM NaH2PO4 buffer (pH 7.0). The target peptides were subsequently eluted using a solution of 5% ACN in 1 M NaCl and 20 mM NaH_2_PO_4_ buffer (pH 7.0). The eluents containing coffeetides were collected and subjected to multiple rounds of preparative reversed-phase high-performance liquid chromatography (RP-HPLC) using a linear gradient ranging from 10% to 60% with buffer A (0.1% TFA in water) and buffer B (0.1% TFA in 100% ACN). This purification process was executed using an Aeris XB-C18 column (particle size 5 µm, dimensions 250 mm × 22 mm; Phenomenex, CA, USA) at a flow rate of 5 mL/min.

Fractions displaying a positive signal within the desired molecular weight range were lyophilized and subjected to further steps involving sulfur reduction and alkylation for sequence determination. Specifically, a 50 mM ammonium bicarbonate buffer (pH 8.0) was introduced to a solution containing 0.2 mg/mL of each peptide, followed by the addition of 50 mM dithiothreitol (DTT). This reduction process was conducted at 37 °C for 1 h, after which S-alkylation was performed using 100 mM iodoacetamide (IAM) at room temperature for 1 h.

### 4.5. Data Mining and Bioinformatics Analysis

The translated nucleotide Basic Local Alignment Search Tool (tBLASTn) was employed to conduct a search for sequence homologs of coffeetides. The ExPaSy translation tool [70] was utilized to perform translation on all EST sequence findings. For the determination of open reading frames, we defined the region spanning from the specified start codon (ATG) to the stop codons (TAA, TAG, and TGA). To identify the cleavage site of the signal peptide, we employed the SignalP 4.0 tool [71]. Predictions for the isoelectric point were generated using the ProtParam tool [72]. The precursor sequences were aligned using Bioedit [73], and the sequence logo was constructed using WebLogo [74].

### 4.6. Sequence Determination of Coffeetides

The S-alkylated sample underwent desalination using a C18 Zip-tip, followed by lyophilization and reconstitution in 0.1% formic acid (FA) before being subjected to LC-MS/MS analysis. This analysis was conducted on a Dionex UltiMate 3000 UHPLC system, coupled online with an LTQ Orbitrap Elite mass spectrometer (Thermo Fisher Scientific, Bremen, Germany), equipped with a nanoelectrospray ion source (Bruker-Michrom, Auburn, USA). The elution process involved transitioning from eluent A (0.1% FA) to eluent B (90% CAN/0.1% FA) at a flow rate of 0.3 µL/min.

For the standard data-dependent analysis, LTQ Tune Plus software (Thermo Fisher Scientific, Bremen, Germany) was utilized to configure the Thermo Scientific Orbitrap Elite mass spectrometer in positive ion mode. The nanoelectrospray ion source, specifically Michrom’s Thermo CaptiveSpray, was employed. This method alternated between Full FT-MS scans (350–3000 *m*/*z*, resolution 60,000, with 1 µscan per spectrum) and FT-MS/MS scans employing 65, 80, and 95 ms ETD activation times (110–2000 *m*/*z*, resolution 30,000, with 2 µscan averages per MS/MS spectrum). The selection of precursor ions in the MS scans had a lower threshold set at 5000 counts, and ions with a charge >2+ were isolated within a 2 Da mass isolation window and subjected to fragmentation.

The operational parameters included a source voltage of 1.5 kV and a capillary temperature of 250 °C. The automatic gain control for Full MS and MS2 was set at 1 × 10^6^ and 5 × 10^5^, respectively. Data processing was carried out using PEAKS studio version 7.5 (Bioinformatics Solutions, ON, Canada) with a tolerance of 10 ppm for MS and 0.05 Da for MS/MS. The false discovery rate was maintained at 0.1%.

### 4.7. Disulfide Mapping

Coffeetide cC1a (0.5 mg) underwent a partial reduction process using 20 mM tris(2-carboxyethyl) phosphine (TCEP) in a 100 mM citrate buffer (pH 3.0) solution, totaling 500 µL, and was maintained at 55 °C for 50 min. Following this, N-ethylmaleimide (NEM) powder was introduced into the mixture, achieving a final concentration of 50 mM, and the mixture was incubated at 55 °C for 30 min. To halt the reaction, the mixture was promptly injected into an HPLC system equipped with a C18 column (250 × 4.6 mm).

Through the application of a linear gradient, transitioning from 45% to 60% buffer B, intermediate species were effectively separated. The resulting peaks were collected for further analysis via MALDI-TOF MS to confirm the number of NEM-alkylated cysteines. Subsequently, the NEM-alkylated intermediate species underwent full reduction through treatment with 50 mM DTT, incubated at 37 °C for 1 h. This was followed by an alkylation step using 100 mM IAM at room temperature for an additional 1 h. To complete the process, the reaction was terminated by injecting the mixture back into the HPLC system. The S-alkylated peptides were then directly subjected to MS/MS sequencing.

### 4.8. NMR Solution Structure

Assignment and structure determinations were performed on a Brucker 800 MHz NMR spectrometer (Bruker, IL, USA). Coffeetide cC1a was dissolved in 500 µL of 30% Deuterated DMSO/70% H_2_O. The concentration of the NMR sample was approximately 1 mM. Nuclear Overhauser effect spectroscopy (NOESY) experiments were performed with mixing times of 200 and 300 ms in collecting NOE spectra for coffeetide [75,76]. Total correlation spectroscopy (TOCSY) data were recorded with a mixing time of 69 or 78 ms using MLEV17 spin lock pulses [77]. The spectrum width was set at 12 ppm and the center was set at 4.375 ppm. The spectrums were analyzed using Bruker TOPSPIN 2.1 or the NMRPipe [78] program on a Linux workstation. All 2D NMR were recorded in the phase-sensitive model using the time-proportional phase increment method [79], with 2048 data points in the t2 domain and 512 points in the t1 domain. The assignment of NOE cross-peaks was determined using Sparky 3.12 software [80]. The proton chemical shift assignments for individual amino acid residues were determined through the utilization of 2D 1H-1H TOCSY and 1H-1H NOESY spectroscopy techniques. Concurrently, the proton-proton distance restraints were acquired by analyzing the intensities of NOE (Nuclear Overhauser Effect) cross-peaks in 2D 1H-1H NOESY experiments. The chemical shifts of protons were referenced to internal sodium 3-(trimethylsilyl)-1-propanesulfonate (DSS-d_6_). The structure calculation was carried out using the software CNSsolve 1.3 [81] and displayed by PyMoL [82]. The determination of the molecular structure involved the incorporation of proton-proton distance restraints and constraints for the three disulfide bonds within a standard simulated annealing protocol. The distance restraints were categorized into three classes, taking into account the intensities of the NOE cross-peaks: (1) strong (1.8 < d < 2.9 Å), (2) medium (1.8 < d < 3.5 Å), and (3) weak (1.8 Å < d < 5 Å). A total of 100 structural conformations were computed, and subsequently, the 10 structures displaying the lowest energy were selected for further analysis and presentation of the data. The structural integrity was assessed using the PROCHECK program. The structural data are accessible under the PDB code 6JI7.

### 4.9. Chemical Synthesis of Coffeetide

Solid-phase peptide synthesis was used to chemically synthesize coffeetide [83]. The synthesis process involved the swelling of 2-chlorotrityl chloride resin (0.09 g, 0.1 mmol) in dichloromethane (DCM) for 30 min. Subsequently, N-alpha-(9-Fluorenylmethyloxycarbonyl)-S-trityl-L-cysteine (Fmoc-L-Cys (Trt)-OH) and benzotriazol-1-yl-oxytripyrrolidinophosphonium hexafluorophosphate (PyBop) (208 mg, 4 eq., 0.4 mmol) were introduced to the resin in the presence of N, N-diisopropylethyamine (DIEA) (174 µL, 4 eq., 0.4 mmol) in N, N-dimethylformamide (DMF). The reaction was conducted at room temperature in a shaker for 1 h and repeated once to ensure the completion of the coupling reaction. Following this, deprotection reactions were carried out using 20% piperidine with 0.1 M N-hydroxybenzotriazole (HOBt) in DMF for two 10-min cycles. The presence of free amines was confirmed using a Kaiser test, which involved a mixture of ninhydrin, potassium cyanide, and phenol (45 µL, 1:1:1, *v*/*v*/*v*). Fmoc deprotection was considered complete when a blue color appeared on the resin.

Peptide elongation was performed using a MW-assisted Liberty Blue™ Automated Microwave Peptide Synthesizer (CEM corporation, NC, USA). Standard protocols were followed with Fmoc amino acids, PyBop, and DIEA (1, 5, and 5 eq., respectively) in DMF for single couplings at 50 °C for 10 min each, except for Cys (Trt) and Arg (Pbf), which underwent coupling at 50 °C for 10 min twice. The final cleavage from the resin and the removal of all side chain protecting groups were achieved through treatment with a mixture consisting of 2.5% triisopropylsilane (TIS), 2.5% H_2_O, 2.5% 1,2-ethanethiol (EDT), and 92.5% trifluoroacetic acid (TFA) for 1 h. The cleaved peptide was then precipitated using diethyl ether (9 eq.) and centrifuged at 6000 rpm for 10 min to obtain crude peptide. Confirmation of the peptide’s presence was achieved through RP-UHPLC analysis, employing a linear gradient of 10% ACN/0.1% TFA over 18 min. Peaks were collected and verified by MALDI-TOF MS. A summary of the synthesis scheme can be found in Supplementary Figure S6.

### 4.10. Oxidative Folding of Coffeetide cC1a

We explored 18 distinct folding conditions by varying redox reagents, reaction times, cosolvents, and redox reagent concentrations. All experiments were conducted within an ammonium bicarbonate buffer at a pH of 8. We employed two pairs of redox reagents: reduced glutathione (GSH)/oxidized glutathione (GSSG) and cysteamine/cystamine. For each specific condition, 0.38 mg of cC1a was dissolved in a total volume of 100 µL of solvent, resulting in a final concentration of 1 mM. To terminate the reaction, 2 M hydrochloric acid was introduced at 24-h intervals.

The folding progress was continually monitored through analytical Reversed Phase-ultra high-performance liquid chromatography (RP-UHPLC), utilizing a gradient ranging from 20% to 80% buffer B over a span of 18 min. The folding yield was quantified by comparing the peak area of cC1a before and after the folding reaction.

### 4.11. Stability Assays

Thermal and acidic stability: A solution of purified 200 µM coffeetide cC1a was subjected to rigorous conditions, including incubation at 100 °C in a water bath and exposure to 2 M hydrochloric acid for 2 h. The reactions were promptly quenched, either by placing the samples in an ice bath for 10 min or by adding 1 M sodium hydroxide at various time intervals (0, 30, 60, 90, 120 min). Subsequently, the treated samples were injected into RP-UHPLC to evaluate the presence of coffeetide cC1a and quantify any degradation. Each experiment was conducted in triplicate, and MALDI-TOF MS was employed to analyze the collected peaks.

Proteolytic stability: In this test, purified 200 µM coffeetide cC1a was exposed to 37 °C for 6 h in the presence of 4 mg/mL of pepsin in a 100 mM sodium citrate buffer (pH 2.5) or 20 U/mL of aminopeptidase I in 20 mM tricine and 0.05% bovine serum albumin (pH 8.0). The peptide-to-enzyme ratio (*w*/*w*) was maintained at either 20:1 or 50:1. At specific time intervals (0, 2, 4, and 6 h), 20 µL of each sample was injected into RP-UHPLC to monitor degradation.

Human serum-mediated stability: To assess stability in a biologically relevant context, purified 200 µg coffeetide cC1a was incubated with 25% human male serum (AB-type) in phenol red-free Dulbecco’s Modified Eagle Medium (DMEM) at 37 °C for 48 h. At defined time points (0 h, 12 h, 24 h, and 48 h), 50 µL of the samples were collected and treated with 100 µL of 95% ethanol to precipitate serum proteins. After incubation at 4 °C for 15 min and centrifugation at 13,000 rpm for 10 min, the extent of degradation was evaluated through RP-UHPLC chromatograms.

### 4.12. Cell Culture

HeLa cells (human cervical cancer cells) and HUVEC-CS (human umbilical vein endothelial cells) were cultured in DMEM (Dulbecco’s Modified Eagle Medium) from Thermo Scientific, supplemented with 10% fetal bovine serum, 100 U/mL of penicillin, and streptomycin. Cell cultures were maintained in a 5% CO_2_ incubator at 37 °C.

### 4.13. Toxicity Assay

To evaluate cell viability, a 3-(4,5-dimethylthiazolyl-2)-2,5-diphenyltetrazolium bromide (MTT) dye reduction assay was employed. In brief, HeLa and HUVEC-CS cells were subjected to treatment with either coffeetide cC1a or 0.1% Triton X-100 (utilized as a positive control) for a duration of 24 h. Following the incubation period, MTT was introduced to the cells to achieve a final concentration of 0.5 mg/mL and incubated for 3 h at 37 °C. The resulting insoluble formazan crystals were subsequently dissolved by the addition of dimethyl sulfoxide, and absorbance was measured at 550 nm using a microplate reader (Tecan Infinite^®^ 200 Pro, Switzerland).

### 4.14. Cell-Penetrating Assay

HUVEC-CS cells were initially seeded in an 8-well chamber slide (ibidi, Martinsried, Germany) and allowed to incubate for 24 h at 37 °C. Following this incubation period, the cells were exposed to 1 µM fluorescently labeled coffeetide cC1a (FAM-cC1a) and left to incubate for an additional 4 h at 37 °C. To visualize the cell nuclei, Hoechst 33342 staining was applied, and live-cell confocal microscopy was conducted using an LSM 980 Confocal Microscope from Zeiss, Germany.

### 4.15. Isothermal Titration Calorimetry (ITC) Assay

Isothermal Titration Calorimetry (ITC) experiments were conducted at 298 K using the MicroCal PEAQ ITC system from Malvern Instruments Ltd., Malvern, UK. MgCl_2_, KCl, CaCl_2_, and FeCl_3_ were dissolved in a 10 mM Tris buffer containing 100 mM NaCl at a pH of 6.3. Similarly, coffeetide cC1a was prepared in the same buffer. In the initial set of experiments, 200 µM of these ions were incrementally titrated into cC1a, involving twenty 2.5 µL injections, while simultaneously measuring the heat changes. Additional experiments were conducted with higher concentrations of KCl, MgCl_2_, and FeCl_3_ interacting with cC1a. Specifically, 400 µM of KCl, MgCl_2_, and FeCl_3_ were titrated into a solution containing 40 µM of cC1a, utilizing twenty 2.5 µL injections, and monitoring the associated heat changes. Control experiments were conducted by titrating ions into a 10 mM Tris (pH 6.3) buffer containing 100 mM NaCl to account for heat changes resulting from dilution. The obtained titration curves were analyzed using MicroCal PEAQ-ITC analytics software (version 1.0.0.1259, Malvern Instruments Ltd.).

### 4.16. Statistical Analysis

Statistical analyses were conducted using GraphPad Version 6.0d (USA). The data underwent one-way analysis of variance (ANOVA) followed by the Newman-Keuls post hoc test for comparisons. Results were presented as mean ± S.E.M., and a significance level of *p* < 0.05 was deemed statistically significant.

## Figures and Tables

**Table 1:** Coffeetide sequences identified from *C. canephora*, *C. liberica*, *C. arabica*, and *C. racemosa*.

Peptide	Species	Amino Acid Sequence	Mass (Da) ^1^	Charge ^2^	PI	Approach ^3^
Loops		1 2 3 4 5 6				
cC1a	*C. canephora*	ZEGECSPLGEPCAGNPWGCCPGCICIWQ-LTDR---CVGNC	3873.1	−3	4.00	T, P
cC1b	*C. canephora*	-EGECSPLGEPCAGNPWGCCPGCICIWQ-LTDR---CVGNC	3764.3	−3	4.00	T, P
cC1c	*C. canephora*	--GECSPLGEPCAGNPWGCCPGCICIWQ-LTDR---CVGNC	3633.4	−2	4.14	T, P
cC2	*C. canephora*	QEGECSPFGKPCRYNPWGCCDSCVCVAT-PADE-GRCLGNC	4145.6	−2	4.51	T
cC3	*C. canephora*	QEGECSPLGKPCKYNPWGCCGSCLCIVDQP-THEGTCVGNC	4206.7	−2	4.83	T
cC4	*C. canephora*	QEGECSPLGKPCRYNPWGCCGSCLCIVDQP-THEGTCVGNC	4234.7	−2	4.83	T
cC5	*C. canephora*	QEGECSALGKPCRYNPSGCCGLCVCVIPDPTDE-GSCIGIC	4067.7	−3	4.18	T
cC6	*C. canephora*	QEVECSPLGEPCAGNPWGCCPGCICIWQ-LTDR---CVGNC	3931.6	−3	4.00	T
cC7	*C. canephora*	QEPSCIPVLGSCVGNPWGCCPGCMCIRQ-LTDR---CHGYC	3976.6	0	6.69	T
cL1a	*C. liberica*	ZEGECSPAGKPCR--PLGCCGACLCIVDHP-THEGTCVGNC	3819.2	−2	5.36	T, P
cL1b	*C. liberica*	-EGECSPAGKPCR--PLGCCGACLCIVDHP-THEGTCVGNC	3708.2	−2	5.36	T, P
cL1c	*C. liberica*	--GECSPAGKPCR--PLGCCGACLCIVDHP-THEGTCVGNC	3579.1	−1	6.01	T, P
cL2	*C. liberica*	--GECSPLGEPCAGNPWGCCPGCICIWQ-LTDR---CIGNC	3649.2	−2	4.14	T, P
cL3	*C. liberica*	GKDTCIGLLESCKDDPWGCCFGCVCLWP--GDL---CRGSC	3834.4	−2	4.36	T
cL4	*C. liberica*	QEGECSPAGKSCR--PVRCCDFCVCVVDYP-THVGTCRGNC	4069.7	−2	4.36	T
cL5	*C. liberica*	QEGECSPAGKPCR--PVRCCDFCVCVVDYP-THVGTCRGNC	4082.7	0	6.70	T
cL6	*C. liberica*	QEPSCIPVLGSCVGNPWGCCPGCMCIRQ-LTDR---CHGYC	3976.7	0	6.69	T
cA1	*C. arabica*	QEGECSPLGEPCAGNPWGCCPGCICIWQ-LTDR---CIGNC	3903.5	−3	4.00	T
cA2	*C. arabica*	QEGECSPLGEACAGNPWGCCPGCICIWQ-LTDR---CVGNC	3863.5	−3	4.00	T
cA3	*C. arabica*	QEPSCLPAGESCTGNPWGCCPGCICIWQ-LTER---CVGNC	3905.6	−2	4.25	T
cA4	*C. arabica*	QEPSCIPVGEPCAGNPGGCCDGCICIWQ-LTDR---CAGSC	3733.5	−3	3.92	T
cA5	*C. arabica*	QEGECSPLGKPCRYNPRGCCDFCVCVVADVTDEEGSCRGNC	4389.7	−3	4.44	T
cA6	*C. arabica*	RKDTCIGLLESCKDDPYGCCPGCVCLWP--GDL---CRGDC	3884.6	−2	4.46	T
cA7	*C. arabica*	QEGECSPAGKPCR--PVRCCDSCLCIVDYP-THVGTCRGNC	4047.7	0	6.70	T
cA8	*C. arabica*	QEGECSPLGKPCAGNPWGCCPGCICIWQ-LTDR---CIGNC	3902.5	−1	4.68	T
cA9	*C. arabica*	QEPSCIPVGEPCAGNPGGCCDGCICIWQ-LTDR---CAGSC	3733.3	−3	3.92	T
cA10	*C. arabica*	QEGECSPLGEPCAGNPWGCCPGCICIWQ-LTDR---CVGNC	3890.1	−3	4.00	T
cR1	*C. racemosa*	QEPRCIPALGSCVGNPWGCCFGCMCIRQ-LTDR---CLGYC	4043.7	+1	7.70	T
cR2	*C. racemosa*	QEPRCIPVFGSCVGNPWGCCFGCMCIRQ-HTNR---CLGYC	4128.7	+2	8.22	T
cR3	*C. racemosa*	QEGECSPFGKPCRYNPWGCCGSCLCVVDHP-THEGTCVGNC	4263.7	−2	5.36	T
cR4	*C. racemosa*	QEGECSPFGKPCRYNPWGCCGSCVCVVDHP-THEGTCLGNC	4263.7	−2	5.36	T
cR5	*C. racemosa*	QEGKCSPAGKPC--DPWGCCDFCVCVVDFPGGE-GRCAGNC	3885.5	−2	4.44	T
cR6	*C. racemosa*	QEEKCSPAGKPCRYNPRGCCDFCVCVVDFPGGE-GSCLGNC	4218.7	−1	4.94	T
cR7	*C. racemosa*	GKDTCIGLLESCKDDPWGCCPGCVCLWP--GDL---CRGSC	3780.5	−2	4.36	T

^1^ Mass (Da) = calculated mass. ^2^ Charge: the total charge is the sum of positive (lysine, arginine, and histidine residues) and negative (glutamate and aspartate residues) charges present in each sequence. ^3^ Method: the primary sequence was obtained by transcriptomic (T) and/or proteomic (P) approach. The Cys are highlighted in yellow. The assignment of isobaric amino acids such as Leu/Ile was confirmed by the transcriptome. All of the coffeetides contain eight cysteine residues, which are highlighted in yellow. Z = pyroGlu.

## Data Availability

The datasets presented in this study can be found in online repositories. Accession codes of coffeetide cC1a: PDB ID 6JI7 and ginsentide TP1: 2ML7. Genbank accession number: cc1 (DV676066.1), cc2 (DV678117.1), cc3 (DV688598.1), cc4 (DV704915.1), cc5 (DV687022.1), cc6 (DV678112.1), cc7 (DV672477.1), cc8 (DV674842.1), ca1 (GT701156.1), ca2 (GT020922.1), ca3 (GR983294.1), ca4 (GT673445.1), ca5 (GT021473.1), ca6 (GT021132.1), ca7 (GW468514.1), ca8 (GW486522.1), ca9 (GT692623.1), ca10 (GT021252.1), cr1 (GT669255.1), cr2 (GT665740.1), cr3 (GT664847.1), cr4 (GT665189.1), cr5 (GT666921.1), cr6 (GT666030.1), cr7 (GT668698.1), tc2 (CU471503.1), pd1 (CA821362.1), pe1 (AJ780051.1), ptt1 (BU827525.1), pt1 (CA925073.1), as1 (DY543302.1), gh1 (CO491697.1), osj1 (CI251708.1) and ta1 (CK209254.1). OneKp accession number: hg1 (OKEF-2088352), ms1 (zAJFN-2096758) and eco1 (CXSJ-2102568). The transcriptome data of ginsentides used are listed as follows: *Panax notoginseng* flower (SRX378878), *Panax quinquefolius* flower (SRX062267), *Panax ginseng* flower (SRX181263), *Panax ginseng* flower (SRX378873), *Panax notoginseng* leaf (SRX378880), *Panax quinquefolius* seed (SRX529365), *Panax ginseng* root (ERX137460).

## Supplementary Materials

The following supporting information can be downloaded at: https://www.mdpi.com/article/10.3390/molecules28186556/s1, Supplementary data on mass spectrometry, sequencing, oxidative folding, HPLC, and NMR can be found in the supplementary data file. Figures S1–S7: Mass spectrometry profile of coffeetide cC1a before and after *S*-reduction and *S*-alkylation using DTT and IAM, respectively; Mass spectrometry profile of coffeetides cL1a, cL1b, cL1c, and cL2 before and after *S*-reduction and *S*-alkylation using DTT and IAM, respectively; *De novo* sequencing of coffeetides cC1a, cC1b, and cC1c; *De novo* sequencing of coffeetides cL1a, cL1b, cL1c, and cL2; Overlapped 2D NOESY spectra of native (red) and synthetic cC1a (green) displayed by Sparky 3; Synthesis scheme of coffeetide cC1a; Selected different oxidative folding conditions; Tables S1–S3: Oxidative folding conditions for synthetic coffeetide cC1a; Structural statistics for the final 10 conformers of cC1a; Proton chemical shift assignments for each amino acid residues of coffeetide cC1a.

## Sample Availability

Samples of the compounds are available from the authors.
