# Supplementary material for: Ginsentide-like Coffeetides Isolated from Coffee Waste Are Cell-Penetrating and Metal-Binding Microproteins
Source: Molecules. 2023 Sep 10;28(18):6556. doi: 10.3390/molecules28186556 (PMC10538209; doi:10.3390/molecules28186556)
Supplement: Supplementary file 1 [file molecules-28-06556-s001.zip › molecules-2578219-supplementary.pdf]

Ginsentide-like Coffeetides Isolated from Coffee Waste Are Cell-Penetrating and Metal-Binding Microproteins

James P. Tam [1,*], Jiayi Huang [1], Shining Loo [1,2], Yimeng Li [1,3] and Antony Kam [1,4]

[1] Synthetic Enzymes and Natural Products Center, School of Biological Sciences, Nanyang Technological University, 60 Nanyang Drive, Singapore 637551, Singapore; huan0273@e.ntu.edu.sg (J.H.); shining.loo@xjtlu.edu.cn (S.L.); leahlym@163.com (Y.L.); antony.kam@xjtlu.edu.cn (A.K.)
[2] Academy of Pharmacy, Xi'an Jiaotong-Liverpool University, Suzhou 215123, China
[3] School of Food Science and Engineering, South China University of Technology, Guangzhou 510640, China
[4] Department of Biological Sciences, Xi'an Jiaotong-Liverpool University, Suzhou 215123, China
* Correspondence: jptam@ntu.edu.sg

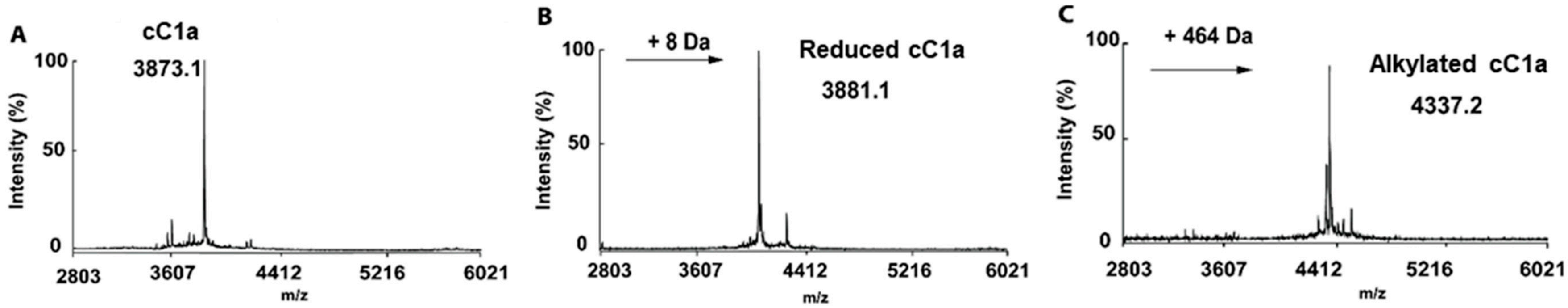

Figure S1. Mass spectrometry profile of coffeetide cC1a before and after *S*-reduction and *S*-alkylation using DTT and IAM, respectively. (A) cC1a, (B) *S*-reduced cC1a by DTT showing an increase of 8 Da, (C) *S*-alkylated cC1a by IAM showing an increase of 464 Da, indicating that coffeetide cC1a is an 8-Cys-microprotein.

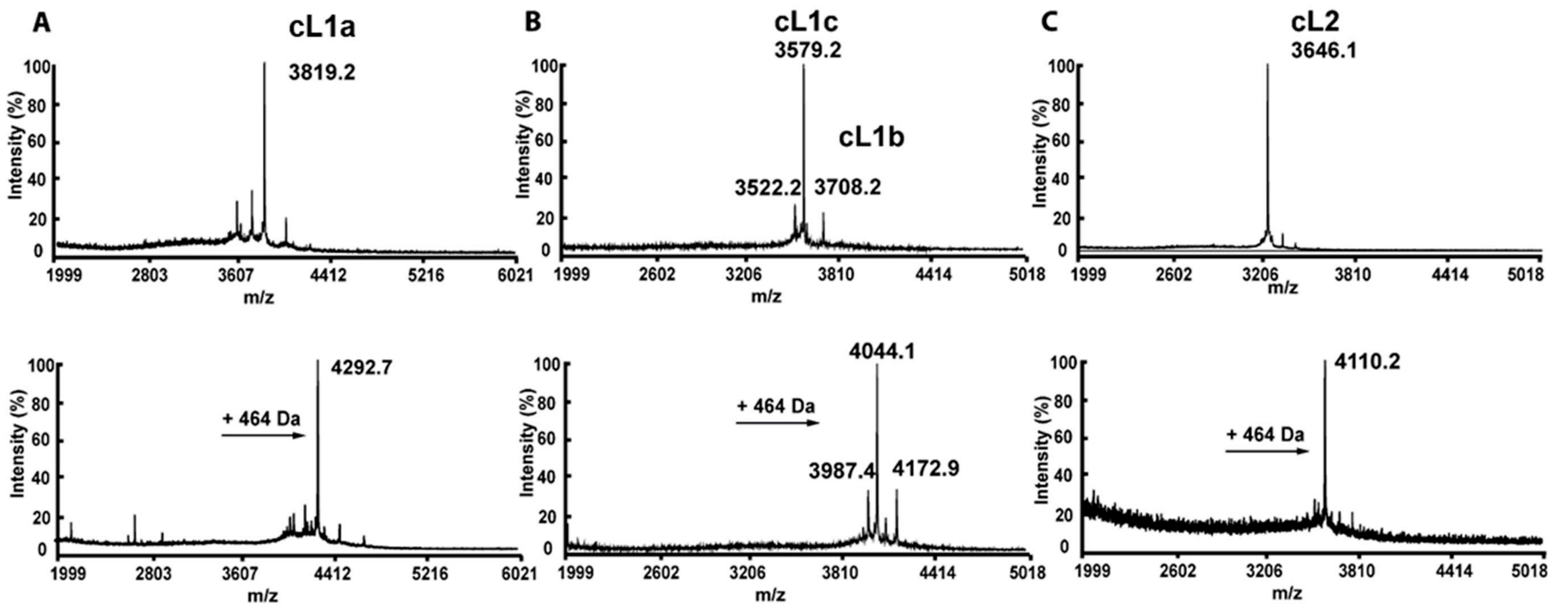

Figure S2. Mass spectrometry profile of coffeetides cL1a, cL1b, cL1c, and cL2 before and after *S*-reduction and *S*-alkylation using DTT and IAM, respectively. (A) cL1a and alkylated cL1a, (B) cL1b and cL1c, and alkylated cL1b and alkylated cL1c (C) cL2 and alkylated cL1. All coffeetides cL1a, cL1b, cL1c, and cL2 after *S*-alkylation with IAM showed an increase of 464 Da, indicating the presence of eight cysteine residues.

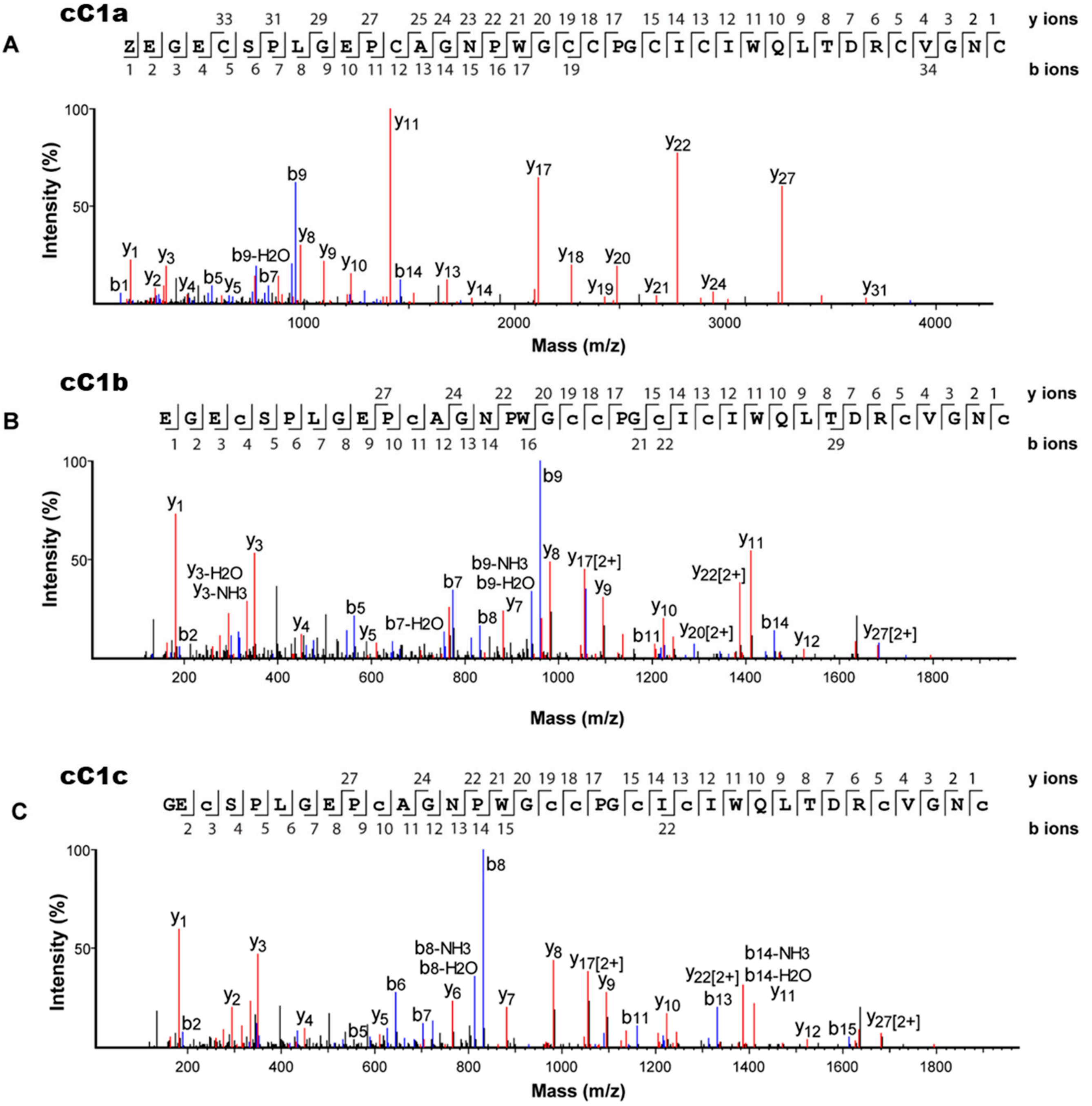

Figure S3. *De novo* sequencing of coffeetides cC1a, cC1b, and cC1c. The sequences of the fragments were deduced using the *b*-ions and *y*-ions generated from LC-ESI-LTQ-Orbitrap MS/MS in positive ion mode, Assignment of isobaric amino acids such as Leu/IIe were confirmed using transcriptomic analysis. (A) cC1a, (B) cC1b, and (C) cC1c.

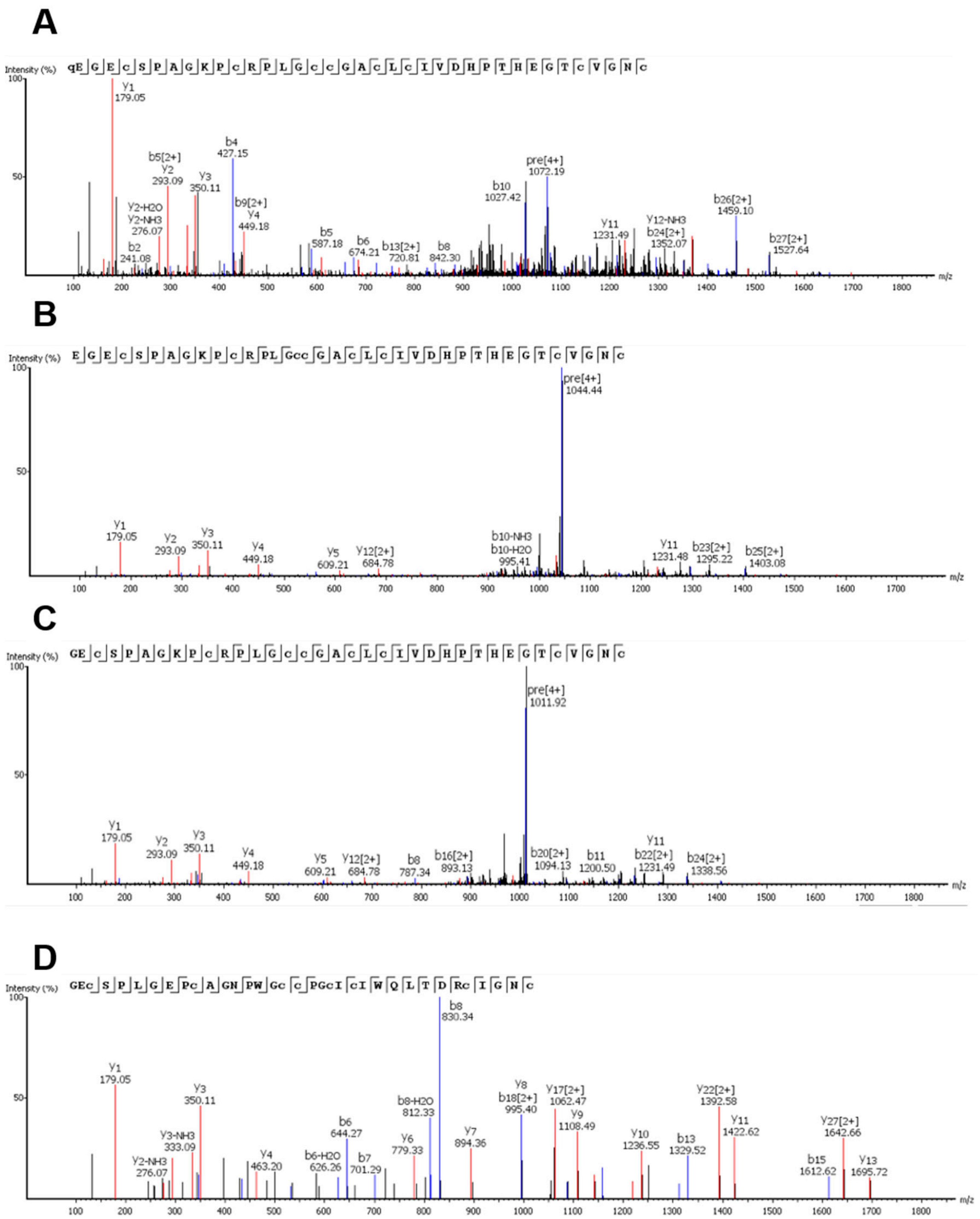

Figure S4. ***De novo*** **sequencing of coffeetides cL1a, cL1b, cL1c, and cL2**. The sequences of the fragments were deduced using the *b*-ions and *y*-ions generated from LC-ESI-LTQ-Orbitrap MS/MS in positive ion mode. Assignment of isobaric amino acids such as Leu/IIe were confirmed using transcriptomic analysis. (A) cL1a, (B) cL1b, (C) cL1c, sand (D) cL2.

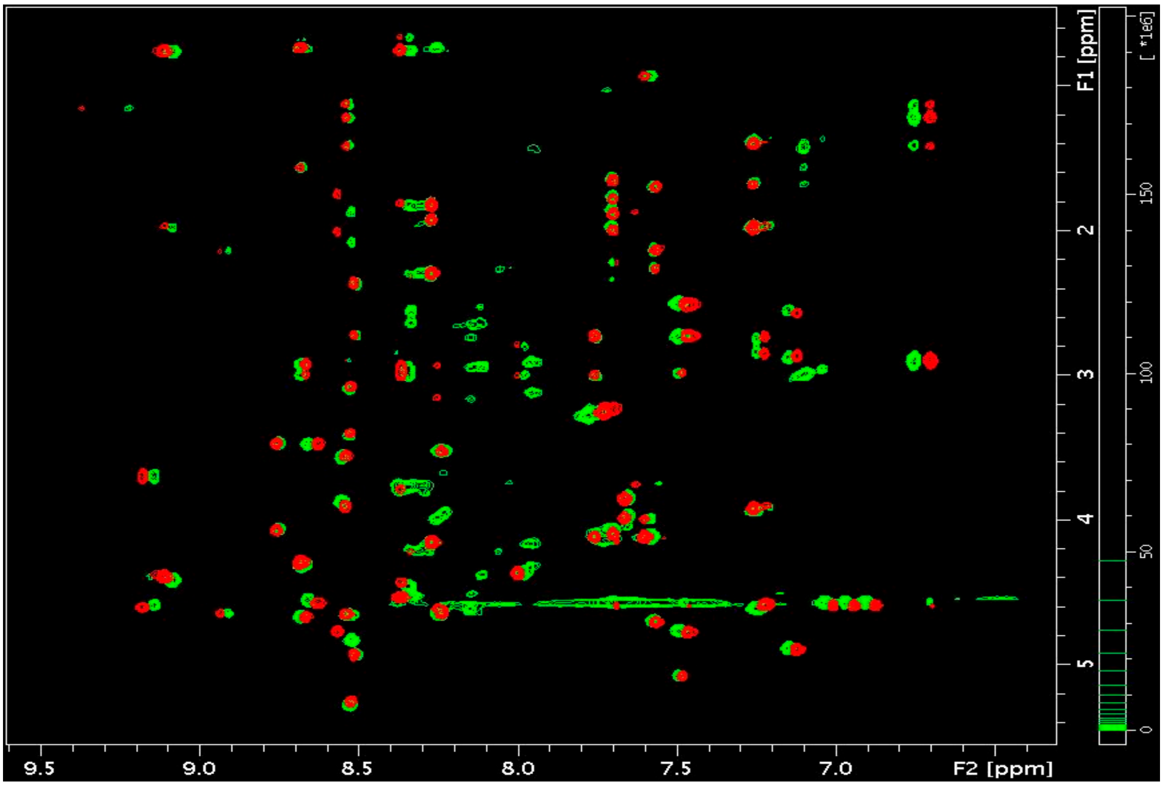

Figure S5. Overlapped 2D NOESY spectra of native (red) and synthetic cC1a (green) displayed by Sparky 3.

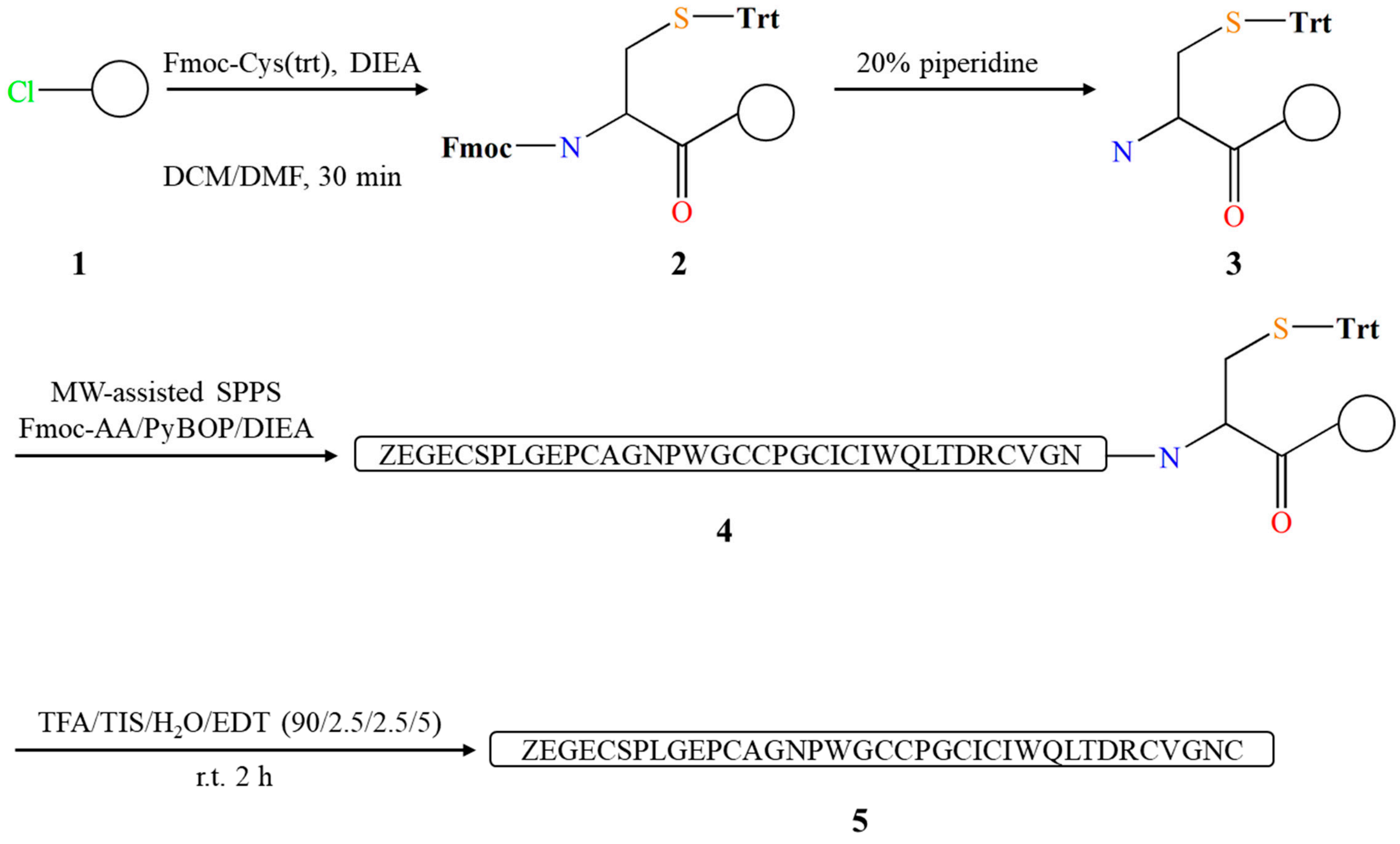

Figure S6. Synthesis scheme of coffeetide cC1a. Coffeetide cC1a was synthesized through solid-phase peptide synthesis using Fmoc-chemistry. Chemical modification of resins (steps1-3) was performed manually, and the subsequent elongation of the peptide (step 4) was performed using an automated microwave peptide synthesizer (CEM Liberty Blue). TFA/TIS/H_2O/EDT with ratio of 90/2.5/2.5/5 was used for final cleavage and deprotection off the resin support before adding ice-cold diethyl ether to precipitate crude linear peptide.

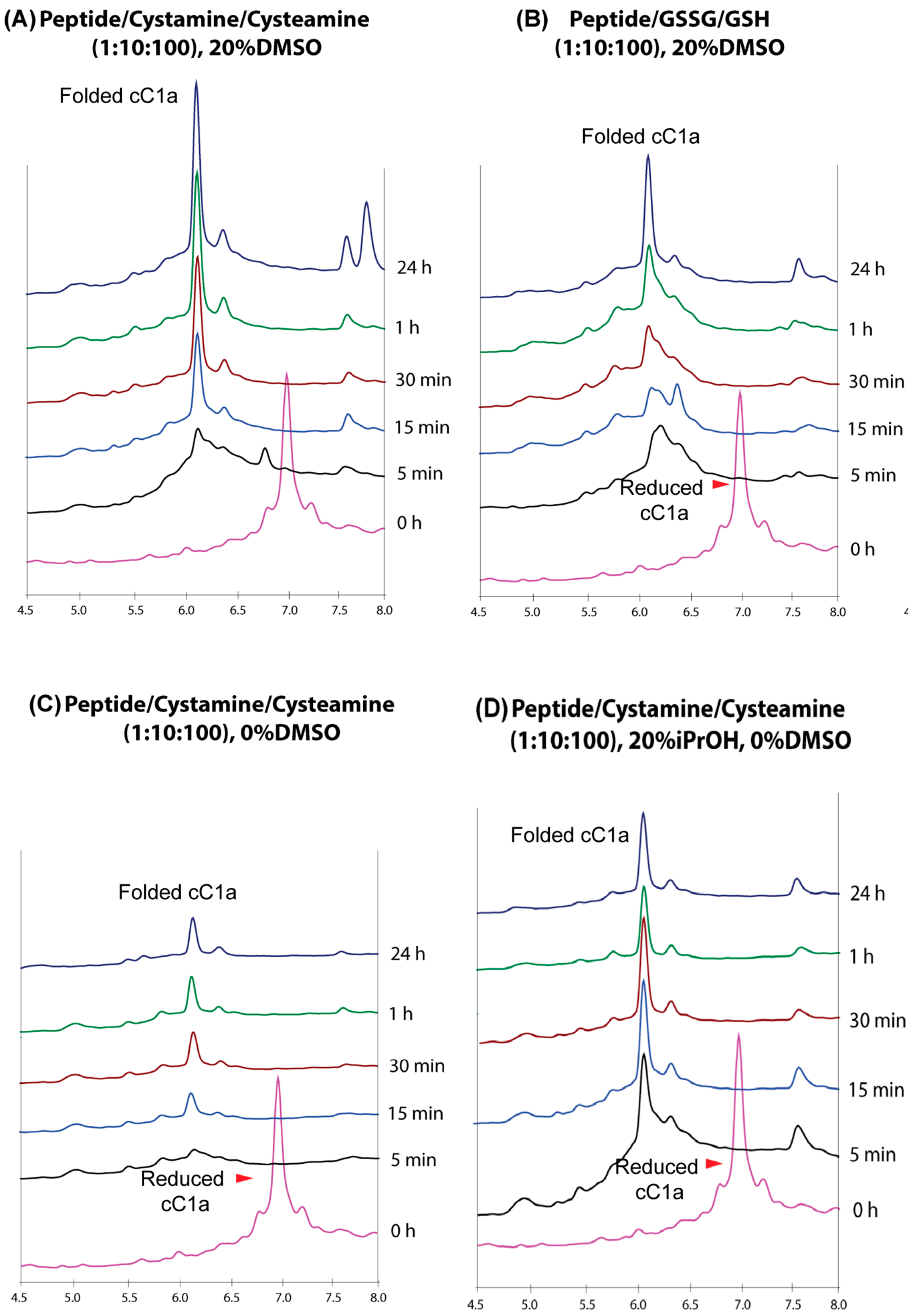

Figure S7. Selected different oxidative folding conditions. The synthetic cC1a was folded with the general folding condition (0.1 M NH_4HCO_3, pH 8). (A) Folding condition: 1 mM peptide+ 10 mM Cystamine/100 mM Cysteamine, with 20% DMSO. (B) Folding condition: 1 mM peptide+ 10 mM GSSG/100mM GSH, with 20% DMSO. (C) Folding condition: 1 mM peptide+ 10 mM Cystamine/100 mM Cysteamine. (D) Folding condition: 1 mM peptide+ 10 mM Cystamine/100 mM Cysteamine, with 20% iProH.

Table S1. Oxidative folding conditions for synthetic coffeetide cC1a

Run	Cystamine (mM)	Cysteamine (mM)	DMSO (%)	IPrOH (%)	Time (h)	Yield (%)
2	10 (GSSG)	100 (GSH)	0	0	24	9
1	10	100	0	0	24	18
3	10	100	10	0	24	59
4	10	100	20	0	24	81
5	10	100	30	0	24	81
6	10	100	20	20	24	29
7	10	100	20	30	24	11
8	20	100	20	0	1	67
9	20	100	20	0	24	77
10	10	200	20	0	1	75
11	10	200	20	0	3	82
12	10	200	20	0	24	82
13	10	300	20	0	1	70
14	10	300	20	0	3	76
15	10	300	20	0	24	78
16	10	400	20	0	1	46
17	10	400	20	0	3	43
18	10	400	20	0	24	42

Table S2. Structural statistics for the final 10 conformers of cC1a[a]

Distance restraints	
Intra-residue (i-j = 0)	89
Sequential (|i-j| = 1)	80
Medium range (2 ≤ |i-j| ≤ 4)	19
Long range (|i-j| ≥ 5)	28
Hydrogen bond	4
Total	220
Average rmsd to the mean structure (Å)[b]	
Backbone atoms	1.10 ± 0.21
Heavy atoms	1.60 ± 0.25
ϕ/ψ space[c]	
Most favored region (%)	56.2
Additionally allowed region (%)	32.5
Generously allowed region (%)	6.7
Disallowed region (%)	4.6
rmsd from covalent geometry	
Bonds (Å)	0.006 ± 0.035
Angles (deg.)	0.210 ± 0.056
Impropers (deg.)	0.030 ± 0.019
rmsd from experimental restraints	
NOEs (Å)	0.0418 ± 0.0089

[a] Selected from 100 calculated conformers according to overall energy.
[b] Calculated with MOLMOL using range 3-13, 18-37.
[c] Calculated with PROCHECK-NMR.

Table S3. Proton chemical shift assignments for each amino acid residues of coffeetide cC1a.

	HN (ppm)	Hα (ppm)	Hβ (ppm)		Others (ppm)
Z1					
E2	8.382	4.297	2.074	1.970	Hγ, 2.441
G3	7.851	3.400			
E4	7.383	4.082	2.125	1.544	Hγ, 1.825
C5	8.102	4.521	3.144	2.942	
S6	9.301	4.753	3.870	3.827	
P7		4.351	2.214	1.963	
L8	7.460	3.502	1.635	1.576	Hγ, 1.417; Hδ, 0.960
G9	8.900	4.226, 3.620			
E10	7.708	4.845	2.278	1.847	Hγ, 2.410
P11		4.580			
C12	8.386	4.749	3.309	3.083	
A13	9.478	3.997	1.301		
G14	8.663	4.043, 3.709			
N15	7.266	4.134	3.007	2.716	
P16		4.134	1.858	1.619	Hγ, 2.056, 1.236
W17	7.888	4.281	3.149	2.881	Hδ1,7.269, Hε1, 10.096
G18	7.800	4.131, 4.003			
C19	8.625	5.085	2.873	2.515	
C20	9.071	4.790	3.387	2.296	
P21		4.346	2.345		
G22	8.748	4.731, 3.619			
C23	8.666	5.398	3.548	3.228	
I24	8.820	4.446	1.712		Hγ: 0.890, 1.400, 1.031; Hδ, 0.813
C25	8.781	4.820	3.143	3.075	
I26	8.504	4.671	1.960		Hγ: 0.902, 1.440, 1.265; Hδ, 0.813
W27	8.473	4.588	3.147	3.087	Hδ1,7.128, Hε1, 10.076
Q28	7.840	4.242	1.927	1.800	Hγ, 2.143, 2.026
L29	7.742	3.904	2.015	1.689	Hδ, 0.915, 0.892
T30	7.739	4.266	4.143		Hγ2, 1.078
D31	8.689	4.926	2.171	1.922	
R32	8.659	4.790	1.565	1.273	Hγ, 1.363
C33	7.363	5.220	3.127	2.634	
V34	9.238	4.549	2.119		Hγ, 0.905, 0.919
G35	8.343	4.781, 3.669			
N36	7.601	4.923	2.876	2.659	
C37	7.357	4.734	2.998	2.880	
